# The Circadian Diet in Age-Related Neurodegeneration: Nutrient Timing, Sleep, and Mitochondrial Mechanisms

**DOI:** 10.3390/nu18213682

**Published:** 2026-11-06

**Authors:** Cristina Manuela Drăgoi, Alina-Crenguţa Nicolae, Ion-Bogdan Dumitrescu

**Affiliations:** 1 Department of Biochemistry, Faculty of Pharmacy, Carol Davila University of Medicine and Pharmacy, 020956 Bucharest, Romania; cristina.dragoi@umfcd.ro; 2 Department of Physics and Informatics, Faculty of Pharmacy, Carol Davila University of Medicine and Pharmacy, 020956 Bucharest, Romania; ion.dumitrescu@umfcd.ro

**Keywords:** circadian rhythms, sleep, neurodegeneration, Alzheimer’s disease, Parkinson’s disease, melatonin, physical activity, glymphatic system, chrononutrition, time-restricted eating, NAD^+^, meal timing, gut microbiota

## Abstract

Circadian rhythms coordinate sleep–wake regulation, endocrine secretion, nutrient handling and cellular energy metabolism. Circadian and sleep disturbances are associated with metabolic and cardiovascular morbidity and with neurodegenerative disorders, particularly Alzheimer’s disease (AD) and Parkinson’s disease (PD), although whether restoring circadian alignment modifies neurodegeneration remains uncertain. This narrative review examines chrononutrition within the broader context of sleep regulation, physical activity and circadian care, distinguishing evidence from cellular and animal models, observational studies and clinical trials. Meal timing and dietary composition act as signals to peripheral clocks, while NAD^+^, sirtuins and AMP-activated protein kinase link nutrient availability with clock regulation and mitochondrial metabolism. We appraise time-restricted eating, protein distribution, NAD^+^ precursors, tryptophan- and melatonin-containing foods, magnesium, omega-3 fatty acids, polyphenols and fermentable fiber. Experimental findings provide mechanistic plausibility, but metabolic improvements and target engagement have not translated into demonstrated neuroprotection. Clinical application must account for levodopa–protein interactions, undernutrition, frailty and sarcopenia. Adequately powered trials with circadian-phase assessment, controlled energy intake and clinically meaningful neurological outcomes are required before a “circadian diet” can be established as a disease-modifying intervention.

## 1. Introduction

Circadian rhythms regulate biological timing across nearly all organ systems, synchronizing physiological processes with environmental cues such as light, temperature, feeding cycles, and social behavior. When these rhythms become desynchronized, due to aging, disease, artificial light exposure, shift work, or behavioral disturbance, multiple pathological consequences arise, including cognitive decline, metabolic dysfunction, tumor development and increased systemic inflammation [1,2,3,4,5,6,7,8,9]. This framework is particularly relevant to older adults with disrupted routines, sleep disorders and progressive difficulty maintaining adequate nutrition. In AD and PD, sleep fragmentation, altered activity rhythms and changes in melatonin secretion are clinically important, but they should not all be equated with a primary disorder of the molecular clock. Human observational studies identify relationships with disease markers and symptoms, whereas experimental manipulations in animals support selected mechanistic links [10,11]. Taken together, human and animal studies are consistent with a bidirectional relationship between circadian disruption and neurodegeneration, although the direction and magnitude of causal effects in humans remain uncertain. Sleep disturbance is also associated with metabolic and cardiovascular risk, and observational data indicate that physical activity modifies the association between poor sleep and mortality [12,13].

In a previous review, we described the molecular interplay among circadian rhythms, chrononutrition, physical training and redox homeostasis in human health in general terms [5]. The present review completes the scientific picture with a series of fundamental aspects. First, it is restricted to age-related neurodegeneration, primarily AD and PD, with supporting discussion of frontotemporal dementia and Huntington’s disease. Second, it appraises the human intervention evidence for specific chrononutritional exposures (time-restricted eating, protein distribution, NAD^+^ precursors, magnesium, tryptophan- and melatonin-containing foods, omega-3 fatty acids and polyphenols), reporting populations, doses, durations and limitations, and it includes negative and recently published trials, such as the phase 3 NOPARK trial of nicotinamide riboside in early PD [14]. Third, it addresses clinical constraints specific to these patients, namely levodopa–protein interactions, undernutrition, frailty and sarcopenia.

### Review Approach and Literature Selection

For this narrative review, the literature was identified through targeted searches of PubMed/MEDLINE and Europe PMC from database inception to October 2026, supplemented by screening of reference lists, publisher pages and disease-specific nutritional and sleep guidelines. Search concepts combined circadian rhythms, sleep, meal timing, time-restricted eating, intermittent fasting, gut microbiota, NAD^+^ precursors, magnesium, tryptophan, melatonin-containing foods, omega-3 fatty acids and polyphenols with Alzheimer’s disease, Parkinson’s disease, mild cognitive impairment, frontotemporal dementia, Huntington’s disease and levodopa–protein interactions. Randomized human trials were prioritized for efficacy statements, and their populations, doses, durations and limitations are reported. Observational studies are used to describe associations, and cellular or animal experiments to describe mechanisms, with the species or experimental system specified. Review articles are cited mainly for context. Because this is a narrative review, no formal risk-of-bias assessment or meta-analysis was performed; the presentation follows the transparency and scientific-reasoning principles of the SANRA scale [15].

## 2. Molecular Basis of Circadian Timekeeping

### Central and Peripheral Timekeeping and Nutrient-Sensitive Clock Regulation

The suprachiasmatic nucleus (SCN) coordinates circadian timing across a distributed network of cellular oscillators. Light information conveyed by melanopsin-expressing intrinsically photosensitive retinal ganglion cells entrains the SCN to the environmental light–dark cycle. Through neural and endocrine outputs, the central pacemaker contributes to the temporal organization of sleep–wake behavior, body temperature and metabolic physiology. However, peripheral oscillators also respond to feeding-related signals, allowing their phase to change without a corresponding shift in the SCN. In mice, Damiola and colleagues demonstrated that temporally restricted feeding shifted peripheral clock-gene expression while leaving SCN rhythmicity largely unchanged. This experimental dissociation provides a mechanistic basis for chrononutrition: meal timing can modify the temporal relationship between central and peripheral oscillators, rather than uniformly resetting the entire circadian system [16].

At the cellular level, circadian oscillations arise from interlocking transcription–translation feedback loops. CLOCK and brain and muscle ARNT-like protein 1 (BMAL1) form a transcriptional activator complex that promotes expression of Period and Cryptochrome genes. Accumulating PER and CRY proteins subsequently inhibit CLOCK–BMAL1-dependent transcription, generating delayed negative feedback. An auxiliary loop regulates BMAL1 expression through the opposing activities of REV-ERB nuclear receptors, which repress transcription, and retinoic acid receptor-related orphan receptors (RORs), which activate it. Thus, REV-ERB and ROR do not simply “stabilize” BMAL1 expression; their antagonistic actions help organize its rhythmic transcription. These mechanisms, characterized through cellular assays and mouse genetic studies, provide multiple points at which metabolic signals can influence clock phase and amplitude [17].

Feeding signals interact with this molecular machinery through distinct post-translational and translational mechanisms. In mouse fibroblasts, AMP-activated protein kinase (AMPK) phosphorylated CRY1 and promoted its degradation, directly linking cellular energy sensing to the stability of a clock repressor. Conversely, Crosby and colleagues demonstrated in cellular and mouse models that insulin and insulin-like growth factor 1 (IGF-1) increased PERIOD protein synthesis and shifted circadian timing. This response involved mechanistic target of rapamycin complex 1 (mTORC1), phosphoinositide signaling and microRNA regulation. These findings indicate that feeding-related hormones and energy-sensitive kinases influence specific components of the oscillator; they do not support treating AMPK, insulin and mTOR as interchangeable mediators of circadian regulation. The direction and magnitude of resetting depend on the timing of the signal and the responsive cellular system [18].

Nicotinamide adenine dinucleotide (NAD^+^) provides a further connection between clock function and metabolic state. Mouse and cellular experiments established that CLOCK–BMAL1 regulates nicotinamide phosphoribosyl transferase (NAMPT), a rate-limiting enzyme in the NAD^+^ salvage pathway. Rhythmic NAD^+^ availability, in turn, influences the activity of the NAD^+^-dependent deacetylase sirtuin 1 (SIRT1), which feeds back on clock-associated transcription. A complementary mitochondrial mechanism was demonstrated in mice by Peek and colleagues, who linked circadian NAD^+^ availability to SIRT3-dependent protein deacetylation and mitochondrial oxidative metabolism. Distinguishing these nuclear and mitochondrial processes is important: evidence that NAD^+^ couples clock regulation to peripheral oxidative metabolism does not establish that dietary NAD^+^ precursors restore neuronal clock function or prevent neurodegeneration in humans [19,20,21].

Human experimental evidence supports tissue-specific responsiveness to meal timing. In a controlled study of ten healthy men, delaying meals by five hours delayed the plasma glucose rhythm by approximately 5.7 h and the rhythm of adipose-tissue PER2 expression by approximately one hour. Plasma melatonin and cortisol rhythms did not shift significantly, and actigraphic sleep measures were not significantly altered. These findings demonstrate that meal timing can influence peripheral metabolic and molecular rhythms without a detectable change in established markers of central circadian phase. They do not establish improved sleep or neurological benefit, and their generalizability to older adults with Alzheimer’s disease or Parkinson’s disease remains uncertain. Accordingly, a shift in peripheral phase should not automatically be interpreted as beneficial circadian realignment. The relevance of clock proteins to neurodegeneration also extends beyond their role in behavioral rhythmicity. In mice, Musiek and colleagues showed that deletion of *Bmal1* in neurons and glia produced oxidative injury, astrogliosis and synaptic pathology despite preserved behavioral and sleep–wake rhythms. This finding indicates that local clock-protein functions involved in cerebral redox homeostasis can be disrupted without overt loss of organism-level rhythmicity. It also limits therapeutic extrapolation: improving meal regularity or sleep timing cannot be assumed to reproduce restoration of BMAL1-dependent cellular functions. Collectively, these mechanisms support investigating chrononutrition as a means of modifying metabolic timing, while leaving its capacity to preserve neuronal function or alter the progression of Alzheimer’s and Parkinson’s diseases as a clinical question requiring direct testing [22,23].

## 3. Circadian and Sleep Disturbances in Neurodegenerative Diseases: The Relevance of Meal Timing and Chrononutrition

### 3.1. Parkinson’s Disease

Parkinson’s disease (PD) is increasingly recognized not only as a neurodegenerative disorder of the dopaminergic system but also as a disease of circadian disruption, in which alterations in biological rhythms emerge years or even decades prior to motor symptom onset. These abnormalities affect sleep, endocrine regulation, autonomic stability, metabolism, and behavior, reflecting widespread impairment of central and peripheral oscillators [24,25]. Actigraphy and rest–activity monitoring consistently show that PD patients exhibit blunted circadian rhythmicity, characterized by reduced variance between daytime activity and nighttime rest. This phenomenon is attributed to degeneration in hypothalamic regions projecting to the SCN, dopaminergic depletion in the basal ganglia, and impaired light responsiveness. Patients typically show delayed or unstable activity peaks, irregular day–night transitions and a lower rhythm amplitude that correlates with disease severity. Rest–activity degradation is also associated with dopaminergic medication fluctuations and worsening non-motor symptoms [24,26,27].

Excessive daytime sleepiness (EDS) is one of the earliest and most prevalent sleep–wake symptoms in PD. Proposed mechanisms include degeneration of wake-promoting nuclei, such as the locus coeruleus and tuberomammillary nucleus, altered orexin (hypocretin) signaling, abnormal circadian modulation of alertness and altered melatonin rhythms; reduced orexin-A concentrations have been reported in PD, although findings vary with the sampling site and disease stage [28]. Up to 60–80% of patients with PD experience difficulties initiating or maintaining sleep. Contributing factors include degeneration of the ventrolateral preoptic nucleus, nocturnal rigidity, tremor and dystonia, altered circadian temperature rhythms and changes in melatonin signaling, and insomnia severity correlates with bradykinesia and anxiety [24,29]. REM sleep behavior disorder (RBD) is characterized by loss of REM atonia and dream-enactment behaviors. Isolated RBD is a strong prodromal marker of synucleinopathy: longitudinal cohorts indicate that most affected individuals eventually develop PD, dementia with Lewy bodies or multiple system atrophy, with a cumulative risk that rises with the duration of follow-up, although published estimates vary between cohorts and depend on the small number of patients followed for the longest intervals [29]. Patients with isolated RBD also show circadian alterations, including reduced melatonin amplitude, before the onset of motor symptoms [29].

Cardiovascular autonomic dysfunction in PD has a marked circadian component. Patients frequently show loss of the normal nocturnal fall in blood pressure (non-dipping), blunted circadian variation in heart rate variability, and supine hypertension combined with orthostatic hypotension. These abnormalities reflect degeneration of central and peripheral autonomic pathways, and loss of nocturnal dipping has been associated with cognitive impairment and faster disease progression [30,31,32,33]. Melatonin rhythms are also altered: nocturnal melatonin secretion is reduced and the circadian melatonin profile is flattened, particularly in patients with excessive daytime sleepiness [10], and changes in melatonin timing have been reported in early PD and in isolated RBD [24,26]. These findings are consistent with impaired SCN output and degeneration of pathways that regulate pineal function. Alterations in clock-gene expression have been described mainly in experimental systems. In rodent models of PD (α-synuclein overexpression and MPTP), studies report loss of rhythmic dopaminergic firing, increased oxidative stress and greater vulnerability of dopaminergic neurons with *Bmal1* deficiency, and altered *Per* gene expression, while clock proteins have been characterized in the substantia nigra of non-human primates [34,35,36,37,38]. Clinically, loss of circadian regulation accompanies motor fluctuations, freezing of gait and early-morning akinesia, and patients often report predictable diurnal patterns of symptom severity [29]. Lower stability of rest–activity rhythms has been associated with poorer attention, executive and visuospatial function and with depression, anxiety and apathy; whether flattened activity rhythms can serve as a biomarker of progression to Parkinson’s disease dementia requires prospective validation [29,39].

Experimental and observational data suggest that circadian dysfunction in PD may be more than a secondary phenomenon, although its causal contribution in humans has not been established. In rodent models, loss of clock-gene function increases oxidative stress and the vulnerability of dopaminergic neurons, and sleep disruption reduces the clearance of extracellular proteins [23,40]. In patients, blunted melatonin rhythms are associated with excessive daytime sleepiness [10], and loss of nocturnal blood-pressure dipping is associated with greater non-motor burden and cognitive decline [33]. Whether these changes accelerate α-synuclein pathology in humans remains to be demonstrated. PD may therefore be viewed as a multisystem disorder in which circadian dysfunction is a clinically relevant and potentially modifiable component [39,41].

Within this context, chrononutrition provides a rationale for investigating how meal timing and nutrient distribution interact with circadian dysfunction and pharmacological treatment in PD. Human experimental evidence shows that meal timing can shift peripheral metabolic rhythms without altering melatonin phase or improving sleep; therefore, peripheral entrainment should not be equated with restoration of central circadian function or neuroprotection [22]. Preliminary PD-specific evidence includes a small randomized trial involving 40 participants, which evaluated a six-month personalized, plant-rich dietary intervention incorporating a 12-h overnight fasting interval and cessation of food intake at least three hours before bedtime. Reported improvements in selected motor and non-motor outcomes warrant further investigation, but the combined changes in dietary composition, timing and nutritional support prevent attribution of benefit specifically to circadian realignment [42]. A more established clinical consideration is the interaction between dietary protein and levodopa: amino acids derived from protein can compete with levodopa transport, contributing to variable treatment responses. In selected patients with motor fluctuations, supervised redistribution of protein toward the evening may improve daytime drug responsiveness while preserving total daily protein intake. This strategy addresses nutrient–drug interactions and should not be interpreted as evidence of clock resetting. Moreover, a universal early eating window may conflict with individualized protein redistribution and medication schedules. Chrononutritional planning must therefore prioritize adequate energy and protein intake and account for dysphagia, gastrointestinal dysfunction, unintentional weight loss and sarcopenia risk [43,44,45]. Accordingly, the “circadian diet” in PD is best framed as an individualized adjunct to nutritional and symptomatic care. Trials that separately assess meal timing and dietary composition, incorporate circadian-phase measurements, and monitor nutritional status alongside neurological outcomes are needed to establish whether any benefits reflect circadian mechanisms or extend beyond symptom management.

### 3.2. Alzheimer’s Disease

Alzheimer’s disease (AD) exhibits some of the most severe and multifaceted circadian disturbances documented across neurological disorders. These abnormalities extend far beyond sleep disruption and reflect widespread degeneration of neural structures essential for rhythmic regulation. Patients with AD demonstrate pronounced breakdown of the sleep–wake cycle. The characteristic features are frequent nocturnal awakenings, reduced total sleep time, excessive daytime sleepiness with increased napping, and loss of consolidated night-time sleep. Actigraphy reveals significantly dampened rest–activity rhythms, with reduced amplitude and weakened circadian organization. These patterns intensify as AD progresses, strongly correlating with cognitive burden and functional decline. Contributing factors include degeneration of sleep-promoting nuclei, loss of SCN output, impaired melatonin secretion and reduced homeostatic sleep pressure. The result is a chronic inability to maintain stable, restorative sleep [46].

“Sundowning”, a syndrome of increased agitation, confusion and behavioral dysregulation in the late afternoon and evening, is a characteristic circadian manifestation of AD. Proposed contributors include weakened SCN-driven consolidation of daytime alertness, reduced tolerance to environmental and sensory stimuli as circadian arousal declines, and altered melatonin signaling and light responsiveness; these mechanisms are derived largely from clinical observation and experimental models and have not been established in prospective studies [47,48]. Neuropathological studies have described loss of SCN neurons in AD, including vasoactive intestinal polypeptide-expressing populations [49]. Because the SCN coordinates circadian timing across the brain and body, its degeneration is expected to reduce circadian amplitude, disorganize melatonin and temperature rhythms, disturb the timing of sleep–wake transitions and diminish responsiveness to zeitgebers such as light. SCN changes may occur early in the disease course, although their timing relative to hippocampal pathology in humans remains uncertain. Slow-wave sleep (SWS) supports memory consolidation and has been linked to the brain’s metabolic clearance systems. In AD, the quantity and quality of SWS decline, slow-wave oscillations become fragmented and sleep spindle activity decreases [50]. In cognitively normal older adults, less SWS has been associated with higher CSF Aβ42 concentrations [51], and preclinical AD, defined by CSF Aβ42, has been associated with poorer sleep efficiency [52]. These human data are associations obtained from CSF measurements; changes in Aβ within brain interstitial fluid have been demonstrated in animal experiments [40]. They therefore do not establish that reduced SWS drives plaque formation or clinical progression in humans. The glymphatic system has been proposed to facilitate the clearance of neurotoxic proteins through convective exchange between cerebrospinal and interstitial fluid. In mice, natural sleep and anaesthesia increased the interstitial space by approximately 60% and accelerated Aβ clearance compared with wakefulness [40]. In living humans, however, glymphatic function can be assessed only indirectly, and its contribution to disease progression remains a mechanistic hypothesis rather than an established pathway. Melatonin secretion is also altered in AD, with reduced night-time concentrations, delayed or absent dim-light melatonin onset, flattened circadian profiles and reduced responsiveness to light [53,54]. In experimental systems, melatonin has antioxidant and neuroprotective properties, and its deficiency has been linked to oxidative stress, mitochondrial vulnerability and impaired synaptic plasticity [55,56,57,58,59,60]; whether reduced melatonin contributes to disease progression in patients has not been established. Collectively, these observations are consistent with a bidirectional relationship. SCN degeneration and sleep fragmentation are associated with AD and may contribute to Aβ and tau accumulation, as suggested by human association studies and animal models, while accumulating pathology can in turn damage structures that generate and transmit circadian signals [40,49,51]. The relative contribution of each direction in humans has not been established, but the association is strong enough to justify evaluating circadian and sleep interventions as adjuncts in AD care.

From a chrononutritional perspective, the interaction between feeding–fasting cycles and circadian regulation provides a rationale for investigating meal timing as an adjunct to nutritional care in AD. Direct mechanistic support comes primarily from animal experiments. In APP23 and APP knock-in mouse models, Whittaker et al. demonstrated that time-restricted feeding improved behavioral rhythmicity and memory, modified hippocampal transcriptional patterns, and reduced amyloid pathology. These findings suggest that the temporal organization of food intake can influence processes relevant to AD, but they do not establish that equivalent interventions restore SCN function or slow neurodegeneration in humans [61]. Human evidence remains preliminary and requires careful differentiation between mild cognitive impairment (MCI) and established AD dementia. In a pilot randomized trial involving 46 older adults with MCI, a 15:9 time-restricted eating intervention over 12 weeks improved selected cognitive outcomes, including the Montreal Cognitive Assessment total score. However, participants did not have dementia, and the investigators could not separate the contribution of meal timing from possible changes in energy intake. Consequently, these results should not be interpreted as evidence of reduced amyloid burden, restored circadian alignment, or delayed progression of AD [62].

For clinical translation, a “circadian diet” should therefore be considered an individualized framework for coordinating meal regularity, nutritional adequacy and daily care, rather than a standardized fasting prescription. Predictable meals coordinated with daytime activity and the patient’s sleep–wake schedule are a reasonable strategy to investigate, but their specific effects on circadian phase, sundowning and cognitive decline require direct evaluation. In patients with established dementia, any restriction of the eating window must be weighed against reduced appetite, feeding difficulties, dysphagia, dehydration and unintentional weight loss. Caregiver involvement and preservation of adequate energy and protein intake take precedence over achieving a predetermined fasting duration. This emphasis is consistent with the ESPEN guideline on nutrition and hydration in dementia, which prioritizes individualized nutritional support and does not recommend routine ketogenic diets or omega-3 supplementation as dementia treatments [63].

### 3.3. Frontotemporal Dementia and Huntington’s Disease

In frontotemporal dementia (FTD), particularly the behavioral variant, hyperphagia, increased preference for sweet foods and impaired satiety link neurodegeneration to altered nutritional behavior. Human studies implicate hypothalamic and reward-related networks, but these findings do not establish that abnormal eating behavior results from circadian misalignment. Chrononutrition therefore offers a hypothesis for investigating whether consistent, caregiver-supported meal schedules can help organize food intake alongside daily sleep–wake routines. Such an approach should prioritize dietary quality and individualized energy requirements; its effects on circadian function, behavioral symptoms and disease progression remain to be established. A “circadian diet” in FTD should consequently be framed as a potential supportive strategy requiring clinical evaluation.

In Huntington’s disease (HD), experimental evidence provides a more direct connection between feeding schedules and circadian regulation. Temporally scheduled feeding restored daily activity patterns and selected hepatic gene-expression rhythms in R6/2 mice [64]. These findings support feeding-related entrainment as a potential therapeutic target, but do not establish clinical efficacy or disease modification in humans. Translation must also account for the substantial nutritional vulnerability of patients with HD, including variable or increased energy requirements, unintentional weight loss and dysphagia [65]. Accordingly, investigation of a “circadian diet” should emphasize consistent meal timing while preserving adequate energy and protein intake, with individualized nutritional monitoring taking precedence over prolonged fasting or restrictive eating windows. Table 1 summarizes the principal circadian, sleep and molecular alterations reported in these disorders.

## 4. Systemic Consequences of Circadian Misalignment

Short sleep and circadian misalignment contribute to impaired glucose tolerance and increased insulin resistance by disrupting the temporal coordination between sleep–wake behavior, feeding–fasting cycles and endogenous metabolic rhythms. Controlled human experiments demonstrate that circadian misalignment can elevate postprandial glucose despite increased insulin concentrations, indicating reduced effectiveness of insulin-mediated glucose regulation. Altered leptin and ghrelin levels may additionally influence appetite and caloric intake, although the magnitude and direction of these hormonal responses depend on sleep restriction, energy balance and experimental conditions. Consequently, adiposity and weight gain should be understood as potential longer-term outcomes of interacting behavioral and metabolic disturbances, rather than inevitable consequences of short-term misalignment. These findings support a role for metabolic pathways as potential mediators of disease risk in chronodisruption. Circadian disruption also affects autonomic function, endothelial reactivity and blood pressure rhythms, providing plausible links between mistimed behavioral cycles and cardiovascular vulnerability. Experimental misalignment increases blood pressure, particularly during scheduled sleep, whereas observational associations between night-shift work and cardiovascular disease cannot be attributed exclusively to circadian mechanisms. The immune system is likewise subject to circadian regulation, and misalignment can alter circulating inflammatory mediators, including interleukin-6 (IL-6) and tumor necrosis factor-α (TNF-α). However, systemic inflammatory changes should not be equated with direct evidence of neuroinflammation, and IL-6 has context-dependent pro- and anti-inflammatory actions. Together, metabolic, autonomic and immune disturbances provide interconnected mechanisms through which chronodisruption may contribute to vascular pathology and neurological vulnerability [66,67,68,69,70,71,72,73,74,75,76]. These consequences also have a chrononutritional dimension: the timing of food intake can modify metabolic responses independently of the sleep schedule. In a controlled simulated night-work experiment, restricting meals to daytime prevented the glucose intolerance observed with nighttime eating, supporting meal timing as a modifiable determinant of metabolic alignment. Nevertheless, this short-term physiological benefit does not establish reduced cardiovascular events or neurodegenerative risk. A “circadian diet” should therefore integrate meal timing with dietary composition, energy balance and individual nutritional requirements, while distinguishing improvements in metabolic regulation from demonstrated disease prevention [77].

### Physical Activity, Sleep, and Longevity: A Synergistic Triad

The UK Biobank study by Huang et al. reported that, among 380,055 participants, the combination of poor sleep and low physical activity was associated with the highest mortality risks: approximately 57% higher all-cause mortality, 67% higher cardiovascular mortality, 45% higher cancer mortality and 91% higher lung cancer mortality. High physical activity attenuated or abolished the associations between poor sleep and mortality. The sleep dimensions associated with mortality included duration, insomnia, snoring, daytime sleepiness and chronotype. These observational findings are consistent with a role for physical activity as a behavioral zeitgeber, although the cohort design does not establish that activity stabilizes circadian rhythms or causally reduces mortality [13,78].

Experimental studies suggest several pathways through which circadian disruption could contribute to neurodegeneration: destabilization of clock-controlled gene expression, sleep disturbances that reduce the clearance of extracellular proteins in rodents, and metabolic changes that increase oxidative stress and impair mitochondrial function [23,40,58]. These pathways are likely to interact rather than to follow a fixed sequence, and their relative importance in human AD and PD has not been established (Table 2).

Such alterations may promote neuroinflammatory responses that increase neuronal vulnerability. In parallel, low physical activity is associated with weaker rest–activity rhythms, higher cardiometabolic risk and higher mortality. These interrelated associations help explain why measures of circadian health and sleep quality predict a wide range of outcomes, although they do not show that correcting circadian disruption alters those outcomes [5,79,80,81].

## 5. Emerging Clinical Interventions

### 5.1. Chronobiological Therapies

#### 5.1.1. Bright-Light Therapy and Coordination of Circadian Timing

Bright-light therapy is a non-pharmacological approach to modifying circadian timing and supporting sleep–wake organization in neurodegenerative disorders, including Parkinson’s disease (PD) and Alzheimer’s disease (AD). Its effects are mediated primarily through intrinsically photosensitive retinal ganglion cells (ipRGCs), which convey photic information to the SCN. The response depends on the timing of exposure relative to endogenous circadian phase, as well as light intensity, spectral composition, duration and prior light exposure. Appropriately timed light can advance or delay circadian phase; consequently, morning exposure should not be regarded as universally optimal without considering the patient’s sleep–wake pattern and therapeutic objective. Clinical studies in PD have reported improvements in excessive daytime sleepiness and selected sleep outcomes, although effects on motor symptoms are less consistent. In AD, responses in sleep consolidation, rest–activity rhythmicity and behavioral symptoms are heterogeneous, limiting claims of uniform efficacy against sundowning or circadian fragmentation [82,83]. From a chrononutritional perspective, light exposure and meal timing represent complementary inputs to circadian organization: light primarily entrains the central pacemaker, whereas feeding-related signals influence peripheral metabolic rhythms. Coordinating daytime light exposure, physical activity and regular meals therefore provides a rationale for multimodal intervention. However, the additional benefit of combining these components must be tested directly, because improvements in sleep or daytime alertness do not necessarily demonstrate synchronization of peripheral clocks or modification of neurodegenerative disease.

#### 5.1.2. Timed Melatonin Administration for Insomnia and REM Sleep Behavior Disorder

Melatonin can influence sleep regulation through both circadian phase-shifting and sleep-promoting effects, whose relative contribution depends on administration time, dose, formulation and the underlying sleep disorder. Its chronobiotic action is mediated through melatonin receptors involved in circadian signaling, and administration should be interpreted relative to biological phase rather than clock time alone. In appropriately selected patients, melatonin may facilitate sleep initiation, but benefits for sleep maintenance and objective sleep measures are variable, particularly in neurodegenerative populations. In PD-associated REM Sleep Behavior Disorder (RBD), melatonin may reduce dream-enactment behaviors; nevertheless, symptomatic improvement should not be equated with consistent restoration of REM atonia. Immediate-release and prolonged-release preparations should also be distinguished, because their pharmacokinetic profiles and supporting clinical evidence differ. Current guidance conditionally supports immediate-release melatonin for secondary RBD. Antioxidant and anti-inflammatory effects provide mechanistic interest, but predominantly experimental evidence for these properties does not establish clinical neuroprotection or delayed disease progression [84,85]. The nutritional relevance extends beyond the tryptophan–serotonin–melatonin biosynthetic pathway: dietary precursor availability does not reproduce the timing or exposure achieved with pharmaceutical melatonin. Moreover, controlled human experiments indicate that the overlap between elevated endogenous melatonin and late food intake can impair glucose tolerance, with susceptibility influenced by variation in the melatonin receptor gene MTNR1B. Thus, meal timing is relevant when evaluating melatonin-based interventions, although these metabolic findings do not establish a universal dosing-to-meal interval for patients with PD or AD [86].

#### 5.1.3. Scheduled Feeding and Time-Restricted Eating to Influence Peripheral Clocks

Scheduled feeding acts as a non-photic *zeitgeber* for peripheral clocks in metabolic tissues, including the liver, skeletal muscle and adipose tissue. Feeding–fasting cycles generate recurring changes in insulin signaling, nutrient availability and cellular energy status that interact with molecular clock regulation. These interactions involve nutrient-sensitive pathways, including AMP-activated protein kinase, mechanistic target of rapamycin and NAD^+^-dependent sirtuins, linking temporal patterns of food intake to metabolic transcription and mitochondrial function. Time-restricted eating (TRE) commonly confines daily energy intake to a consistent eating window, often 8–12 h, but the duration of that window alone does not define circadian alignment. Its placement relative to biological phase, habitual sleep and activity is also relevant. Human experiments demonstrate that meal timing can shift peripheral metabolic rhythms without corresponding changes in melatonin or cortisol timing, emphasizing that peripheral resetting does not necessarily indicate restoration of central circadian function. Although selected studies report improvements in glucose regulation and insulin sensitivity, effects on inflammation and mitochondrial function vary by population and experimental model; benefits cannot automatically be extrapolated to patients with PD or AD [22,87,88,89,90]. Within a “circadian diet” framework, meal timing should therefore be evaluated alongside dietary composition, total energy intake and protein adequacy. Regular, nutritionally adequate meals may consequently be more appropriate than prolonged fasting for vulnerable patients. Trials should distinguish the effects of timing from those of reduced energy intake and assess circadian outcomes alongside nutritional status, treatment adherence and clinically meaningful neurological endpoints.

### 5.2. Behavioral and Environmental Optimization

Evening light, particularly short-wavelength-enriched light, suppresses melatonin secretion and delays circadian phase in a dose-dependent manner, and consensus recommendations advise limiting evening and night-time light exposure [80,83,91]. Whether wearable filters translate this physiological effect into better sleep is uncertain: a Cochrane review found inconsistent results across six randomized trials and rated the evidence for an effect of blue-light-filtering spectacle lenses on sleep quality as very low certainty [92]. Warm, dim evening lighting and a dark, quiet and cool sleeping environment are reasonable low-burden measures, but their specific efficacy in AD and PD has not been established in adequately powered trials.

Physical activity serves as a potent non-photic *zeitgeber* and plays a crucial role in stabilizing circadian rhythms. Morning exercise, in particular, promotes earlier circadian phase alignment, improves alertness during the day, and enhances nocturnal sleep depth. Regular moderate-to-vigorous physical activity increases circadian amplitude, supporting more robust oscillations across central and peripheral clocks. Beyond its chronobiological benefits, physical activity improves cardiovascular, metabolic, and cognitive function, offering strong synergistic protection against the health risks associated with sleep disruption. For individuals with Parkinson’s disease, Alzheimer’s disease, and age-related circadian fragmentation, structured morning exercise programs may produce measurable improvements in both motor and non-motor symptoms [93,94].

Sleep hygiene interventions provide a foundational behavioral framework for promoting restorative sleep. These include maintaining regular sleep–wake schedules, avoiding stimulating activities before bedtime, limiting caffeine and alcohol consumption, and creating a sleep environment free of disruption. Cognitive–behavioral therapy for insomnia (CBT-I) builds on these principles by addressing maladaptive thoughts and behaviors that perpetuate sleep difficulties [95]. Although CBT-I is widely used for chronic insomnia, including in younger populations [96], its efficacy in patients with AD or PD has not been established in adequately powered trials, and cognitive and motor impairment may require adapted, caregiver-supported delivery. Benefits demonstrated in other populations should therefore not be assumed to extend to patients with neurodegenerative disorders.

### 5.3. Precision Circadian Medicine

The emerging field of precision circadian medicine seeks to individualize therapeutic strategies by integrating biological, behavioral, and environmental markers of circadian function. Rather than applying uniform interventions, this approach recognizes the substantial inter-individual variability in circadian timing, sleep architecture, molecular rhythms, and environmental sensitivity. Precision circadian medicine aims to tailor interventions based on measurable biomarkers, enabling more effective prevention and treatment of circadian-related disorders, including neurodegenerative, metabolic, and psychiatric conditions [97,98]. One promising direction is the development of individualized melatonin secretion profiles in order to optimize treatment timing and dosage. Melatonin rhythms vary widely among individuals, particularly in aging populations and patients with Parkinson’s disease or Alzheimer’s disease, where secretion is blunted or phase-shifted. Personalized melatonin assessments—based on serial salivary or plasma sampling—allow clinicians to identify a patient’s specific dim-light melatonin onset (DLMO) and adjust melatonin administration accordingly. This tailored approach may improve therapeutic efficacy for insomnia, circadian phase disorders, and REM Sleep Behavior Disorder (RBD), while minimizing adverse effects and chronobiological misalignment [99,100,101,102,103,104].

#### Clock Gene Expression Biomarkers

Biochemical and genetic biomarkers could help guide treatment strategies, monitor disease progression and identify patients at higher risk of circadian-related metabolic or neurodegenerative deterioration, by analogy with biomarker-guided monitoring in metabolic, hepatic, renal and bone disorders and in sleep-disrupted occupational groups [105,106,107,108,109,110,111,112]. Advances in molecular chronobiology may eventually allow clock gene expression profiles to be used as diagnostic and therapeutic biomarkers. Peripheral tissues such as blood monocytes, buccal cells, or hair follicles exhibit rhythmic expression of core clock genes including BMAL1, PER1/2, CRY1/2, and REV-ERBα. Quantifying these oscillations across time may offer insight into circadian phase, amplitude, and robustness, providing a molecular fingerprint of an individual’s circadian status; experimental studies in rats and in cultured cells further show that clock-gene expression responds to light–dark misalignment and regulates lipid metabolism [112,113,114].

Actigraphy provides an accessible and non-invasive method for assessing real-world circadian patterns. By recording rest–activity cycles over days or weeks, actigraphy allows researchers and clinicians to identify an individual’s chronotype, degree of circadian regularity, sleep efficiency, and amplitude of activity rhythms. Actigraphy-based metrics can inform lifestyle recommendations (e.g., optimal timing for sleep, exercise, and light exposure) and identify circadian phenotypes that respond differently to interventions. For patients with neurodegenerative disease, actigraphy offers invaluable insights into functional circadian deterioration and its relationship to cognitive or motor symptoms [115,116].

Pharmacological modulators of clock proteins remain at a preclinical stage. Pharmacological and genetic modulation of REV-ERB altered the expression of orexigenic genes in experimental models [117], and REV-ERB activation reduced amyloid burden and improved cognitive performance in a mouse model of AD [118]. Structural studies of cryptochrome 1 show how its interaction with CLOCK:BMAL1 determines circadian timing, providing a rationale for compounds that stabilize the repressive phase of the feedback loop [119]. None of these approaches has been tested in clinical trials in neurodegenerative disease, and their therapeutic potential should therefore be regarded as hypothetical.

Table 3 summarizes the main circadian interventions discussed in this section, their principal mechanisms and outcomes, and the type and strength of the supporting evidence. These approaches include photic and non-photic zeitgebers, such as bright-light exposure, melatonin, structured feeding windows and physical activity, as well as pharmacological clock modulators that remain experimental. The evidence is strongest for symptomatic effects on sleep and for metabolic outcomes in populations without neurodegeneration; direct evidence that these interventions modify the course of AD or PD is lacking.

## 6. Chrononutrition and Nutritional Modulation of Circadian and Sleep Health in Neurodegeneration

The interventions discussed above act predominantly on photic and behavioral inputs to the clock. Nutrition provides an additional, modifiable input: the timing and composition of food intake influence peripheral oscillators and nutrient-sensing pathways that interact with the molecular clock [5,21]. This section summarizes the mechanistic basis of chrononutrition; the corresponding human evidence, including its limitations in neurodegenerative populations, is appraised in Section 7.

### 6.1. Meal Timing as a Zeitgeber for Peripheral Clocks

Whereas the suprachiasmatic nucleus is entrained chiefly by light, peripheral clocks in the liver, adipose tissue, skeletal muscle, and gut are entrained predominantly by feeding–fasting cycles. Mistimed food intake, eating during the biological night, uncouples peripheral oscillators from the central pacemaker, producing internal desynchrony that impairs glucose tolerance, lipid handling, and inflammatory tone [5,21,122,123]. In neurodegeneration, such peripheral misalignment may compound central circadian disruption, a hypothesis that has not yet been tested directly.

### 6.2. The NAD^+^–NAMPT–SIRT1 Feedback Loop: A Nutrient-Sensitive Node Linking Feeding, the Clock, and Mitochondrial Function

As outlined in Section 2, the CLOCK–BMAL1–NAMPT–NAD^+^–SIRT1 loop and AMPK signaling connect feeding-dependent energy status with clock regulation and mitochondrial oxidative metabolism, primarily on the basis of mouse and cellular studies [19,20,124,125]. Because NAD^+^ concentrations decline with age in several tissues, NAD^+^ precursors and polyphenolic sirtuin modulators, such as resveratrol and pinostilbene, have been proposed as nutritional means of supporting clock and mitochondrial function [126]. As discussed in Section 7.3, however, clinical trials have so far demonstrated target engagement without clinical benefit in PD or mild cognitive impairment.

### 6.3. Intermittent Fasting: Metabolic and Neuroprotective Potential

Intermittent fasting comprises several regimens, including alternate-day and periodic energy restriction, which are not necessarily aligned with the active phase. Prolonged fasting engages a metabolic switch characterized by lower insulin and mTORC1 signaling, higher AMPK activity, ketogenesis and autophagy; in animal models, these changes have been linked to neuronal stress resistance and proteostasis [123,127,128,129]. These pathways are mechanistically relevant to AD and PD, in which impaired autophagy is implicated in the accumulation of β-amyloid and α-synuclein. Whether such effects occur in patients, and whether daytime-aligned eating enhances nocturnal clearance or redox defense, has not been tested.

### 6.4. Chrono-Active Nutrients Modulating Sleep and Circadian Robustness

Specific dietary constituents influence sleep architecture and circadian signaling through defined biochemistry. Dietary tryptophan supplies the substrate for serotonin and melatonin synthesis, and melatonin acts as a chronobiotic signal and, in experimental systems, as a mitochondrial antioxidant [130]; the limited human evidence for food sources of tryptophan and melatonin is summarized in Section 7.4. Magnesium modulates NMDA-receptor and GABAergic signaling pertinent to sleep continuity, omega-3 fatty acids furnish precursors for specialized pro-resolving mediators that temper neuroinflammation, and polyphenols engage the NAD^+^/sirtuin and AMPK axes [126]; omega-3 supplementation has also been evaluated clinically in other neurological contexts [131]. The temporal distribution of macronutrients is itself a signal: the diurnal patterning of protein intake, for instance, entrains the skeletal-muscle clock and modulates anabolic outcomes [132].

### 6.5. The Gut Microbiota–Short-Chain Fatty Acid Axis as a Circadian and Neuroprotective Interface

The gut microbiota exhibits diurnal oscillations entrained by feeding rhythms and constitutes a further nutritional interface with the circadian and nervous systems. Microbial fermentation of dietary fiber yields short-chain fatty acids that influence host metabolism, immune tone, and the gut–brain axis, whereas sleep deprivation and circadian disruption promote dysbiosis and systemic inflammation [7,133,134]. Diets rich in fermentable fiber and bioactive compounds may therefore support both circadian and neuro-immune homeostasis, positioning the microbiota as a modifiable node within the chrononutritional framework proposed here.

Table 4 summarizes the principal chrononutritional strategies, their biochemical mechanisms, their relevance to circadian, sleep and neurodegenerative outcomes, and the type of supporting evidence.

## 7. Chrononutritional Interventions and Human Evidence

### 7.1. Meal Timing, Time-Restricted Eating and Fasting

Time-restricted eating (TRE) confines daily energy intake to a consistent interval. Intermittent fasting is a broader category that also includes regimens with marked energy restriction on selected days; it is not synonymous with early-day eating. Early TRE generally places intake in the morning and early afternoon in humans. In Sutton and colleagues’ controlled crossover trial, eight men with prediabetes consumed a six-hour eating window, with dinner before 15:00, or a 12-h control schedule for five weeks per condition. Improvements in insulin sensitivity and selected cardiometabolic measures occurred while weight was maintained [120]. This provides metabolic proof of concept, not evidence of a neurological benefit in AD or PD. More pragmatic effects are modest and context-dependent. In the TIMET randomized trial, 108 adults with metabolic syndrome completed three months of standard nutritional care with or without a personalized 8–10-h eating window. The between-group HbA1c difference favored TRE by 0.10 percentage points, with a 95% CI of −0.19 to −0.003 percentage points [121]. Neurological disease progression was not an endpoint. Conversely, metabolic benefits of restricted feeding have also occurred in clock-deficient mice [142]; therefore, improvement after TRE cannot automatically be attributed to restoration of an intact molecular clock.

Disease-relevant evidence is emerging but remains limited. Active-phase restricted feeding improved memory and aspects of amyloid pathology in transgenic AD mouse models [61]. A human pilot randomized trial assigned 46 older adults with mild cognitive impairment to 15:9 TRE or usual intake for 12 weeks and reported short-term improvements in selected cognitive scores [62]. The latter population is not equivalent to biomarker-confirmed AD, and the study did not establish slowed dementia progression or a mitochondrial mechanism. Small samples, practice effects, dietary intake uncertainty and limited follow-up constrain interpretation.

### 7.2. Protein Distribution and Levodopa in Parkinson’s Disease

Meal timing has an established practical relevance in PD that is distinct from speculative disease modification. Dietary amino acids can compete with levodopa transport, including access to the brain, while meals and delayed gastric emptying can affect oral drug delivery. In patients who experience protein-sensitive motor fluctuations, changing the relationship between levodopa and meals, or redistributing protein towards the evening, may improve daytime response. ESPEN’s neurology guideline discusses this approach for selected levodopa-treated patients [43]. This is optimization of symptomatic pharmacotherapy rather than demonstrated resetting of a circadian clock. Protein redistribution should not become indiscriminate protein restriction. The clinical plan should preserve adequate daily protein and energy, account for dysphagia, constipation and appetite, and monitor weight, muscle function and dyskinesia. Increased levodopa effectiveness may require medication review. A predominantly evening protein load can also be impractical when appetite is poor or when evening symptoms interfere with eating. Neurological benefit, nutritional adequacy and feasibility must be evaluated together rather than importing a universal “high-protein breakfast” or prolonged-fasting recommendation.

### 7.3. NAD Precursors and Mitochondrial Target Engagement

Niacin and nicotinamide are nutritional NAD precursors, whereas nicotinamide riboside (NR) and nicotinamide mononucleotide (NMN) have been investigated at supplemental doses. They should not be treated as interchangeable compounds or as established neuroprotective treatments. In NADPARK, 30 newly diagnosed, treatment-naive patients with PD received NR 1000 mg/day or placebo for 30 days. Variable increases in cerebral NAD+ and changes in metabolic and inflammatory measures demonstrated target engagement, while exploratory clinical observations did not establish disease modification [135]. More decisive clinical evidence comes from NOPARK, a phase 3 randomized trial published in October 2026. Among 410 randomized patients with early PD, NR 500 mg twice daily for 52 weeks did not improve clinical outcomes. In the primary analysis of 393 participants, the adjusted between-group difference in the Movement Disorder Society Unified Parkinson Disease Rating Scale parts I–III was 2.72 points (95% CI, 0.47–4.98), favoring placebo. Nonmotor symptom burden also favored placebo, whereas four other key secondary outcomes did not differ significantly. The differences were below the reported thresholds for clinically important change; they do not establish accelerated pathological neurodegeneration. Nevertheless, these findings do not support NR as a disease-modifying treatment for early PD and outweigh inferences of efficacy from short-term target engagement [14].

In a separate ten-week pilot trial involving 20 adults with mild cognitive impairment, NR was escalated to 1000 mg/day. Blood NAD+ increased, but cognition did not improve; exploratory cerebral blood-flow findings did not survive the study’s multiple-comparison caveat [136]. A further randomized crossover study enrolled 46 adults with subjective cognitive decline or mild cognitive impairment (37 completed). NR 1000 mg/day for eight weeks did not improve the primary cognitive outcome, although an exploratory plasma phosphorylated-tau217 signal was reported [143]. This biomarker finding is not evidence of clinical efficacy. In 25 postmenopausal women with prediabetes, NMN 250 mg/day for ten weeks improved muscle insulin sensitivity [144], an outcome outside neurodegeneration. These contrasts show why systemic NAD repletion, cerebral target engagement and clinically meaningful neuroprotection must remain separate claims. Timing relative to circadian phase was not established as an efficacy determinant in these trials.

### 7.4. Magnesium, Tryptophan and Melatonin-Containing Foods

Small trials provide limited sleep evidence for selected nutritional preparations. In 46 older adults with primary insomnia, magnesium 500 mg/day for eight weeks improved several subjective sleep measures compared with placebo [139]. This was not a trial in AD or PD, and its findings do not establish correction of a circadian phase disorder. Supplemental magnesium also requires attention to formulation, renal function, gastrointestinal effects and medication use. A supplement dose studied in a small insomnia trial should not be converted into a routine recommendation for all older patients.

A three-week study of tryptophan-containing preparations reported sleep improvements, with 49 of 57 participants completing the protocol [137]. Its food-source preparation combined approximately 250 mg tryptophan with carbohydrate, making it inappropriate to infer the same effect from any tryptophan-rich meal. A small crossover study of tart cherry juice used 240 mL twice daily for two weeks; eight participants completed the study after sleep-apnea exclusions, and some sleep outcomes improved [138]. The preparation contains multiple bioactive compounds, so its effects cannot be assigned specifically to melatonin. Neither trial established a benefit in a neurodegenerative population, and their doses describe research exposures rather than clinical prescriptions. These food-based findings should also be separated from pharmaceutical melatonin. Endogenous synthesis is controlled by light–dark and neural signals, while exogenous melatonin can act as a chronobiotic signal or phase-shifting agent. The response depends on formulation, dose, biological timing and the clinical indication. Evidence for drug treatment of RBD or insomnia cannot be transferred directly to melatonin-containing foods.

### 7.5. Omega-3 Fatty Acids, Polyphenols and Dietary Patterns

Mechanistic anti-inflammatory or antioxidant activity does not guarantee clinical benefit. In a trial of 402 participants with mild-to-moderate AD, DHA 2 g/day for 18 months did not slow overall cognitive or functional decline compared with placebo [140]. In a 52-week AD trial involving 119 participants, resveratrol escalated from 500 mg/day to 1000 mg twice daily altered some biomarker trajectories, but the trial did not establish disease-modifying efficacy; gastrointestinal symptoms and weight loss were relevant adverse effects [141]. Neither intervention tested individualized circadian dosing.

Dietary fiber and microbiome studies provide a rationale for investigating diet quality and regularity, rather than evidence for a disease-specific “chrono-active” nutrient prescription. In dementia care, adequate intake, hydration, assistance with eating and correction of nutritional problems remain more directly actionable than supplemental neuroprotection claims. The 2024 ESPEN dementia guideline does not recommend routine omega-3 supplementation to prevent cognitive decline [63].

Table 5 presents representative human studies with populations, exposures, outcomes and limitations. It separates direct AD/PD evidence from findings in mild cognitive impairment or other populations. The descriptions of evidence strength are qualitative assessments of directness and design.

## 8. Conclusions

Circadian disruption is implicated in the interactions between neurodegeneration, metabolic dysfunction, immune dysregulation and cardiovascular disease. Experimental studies identify alterations in molecular clock regulation, mitochondrial metabolism, redox homeostasis and inflammatory signaling as potential contributors to neuronal vulnerability. Sleep-dependent protein clearance provides an additional mechanistic hypothesis, although its contribution to human disease progression remains incompletely established. These findings support a bidirectional relationship between circadian dysfunction and neurodegenerative pathology, rather than a uniform causal sequence. Observational associations with morbidity and mortality further emphasize the clinical relevance of circadian health but do not establish that correcting misalignment reduces these risks.

Nutrition offers a modifiable interface between circadian timing and cellular metabolism. Feeding–fasting cycles influence peripheral oscillators, while nutrient-sensitive pathways, including NAD^+^–SIRT1 and AMPK signaling, connect energy availability with clock regulation and mitochondrial function. However, mechanistic plausibility, metabolic improvements and biochemical target engagement should be distinguished from demonstrated neuroprotection. Evidence for time-restricted eating, NAD^+^ precursors and specific dietary constituents remains heterogeneous, with limited support for disease-modifying effects in neurodegenerative populations. Chrononutritional interventions must therefore preserve nutritional adequacy and account for frailty, unintentional weight loss, sarcopenia and nutrient–drug interactions.

An integrated approach combining individualized nutritional care with appropriately timed light exposure, physical activity and sleep interventions warrants further investigation. Melatonin may provide symptomatic benefit in selected sleep disorders, but its effects depend on the indication, timing and formulation. Future trials should incorporate validated circadian-phase measurements, objective sleep assessments, controlled or carefully quantified energy intake, and clinically meaningful neurological outcomes. Until such evidence is available, a “circadian diet” is best regarded as a framework for investigating supportive nutritional care, rather than as an established strategy for preventing neurodegeneration or reducing mortality.

## Figures and Tables

**Table 1:** Circadian and Sleep Disturbances in Neurodegenerative Diseases.

*Disorder*	Circadian Findings	Sleep Disturbances	Molecular Alterations
*Alzheimer’s* *Disease*	Fragmented rhythms; SCN neuron loss	Sundowning, insomnia	↓ Bmal1, Per1/2; glymphatic dysfunction
*Parkinson’s* *Disease*	↓ Melatonin; ↓ amplitude	RBD, insomnia	↓ Bmal1, Per2; dopaminergic degeneration
*Frontotemporal* *Dementia*	Irregular sleep–wake patterns	Nighttime activity	Hypothalamic dysfunction
*Huntington’s* *Disease*	Severe circadian disruption	Insomnia	Loss of clock gene rhythmicity

**Table 2:** Systemic Associated Clinical Conditions of Circadian Disruption.

*Domain*	Mechanisms	Associated Clinical Conditions
*Metabolic*	Insulin resistance, hormonal dysregulation	Obesity, Type 2 Diabetes Mellitus
*Cardiovascular*	Loss of blood pressure dipping, endothelial dysfunction	Hypertension, cardiovascular diseases
*Neurological*	↓ Glymphatic clearance, ↑ aggregation	Alzheimer Disease, Parkinson Disease
*Immune*	↑ IL-6, TNF-α	Chronic inflammation
*Endocrine*	Altered cortisol/melatonin	Fatigue, mood disorders

**Table 3:** Circadian interventions: mechanisms, principal outcomes and type of supporting evidence.

*Intervention*	Mechanism	Principal Outcomes	Type and Strength of Evidence in AD/PD
*Bright-light therapy (timed to circadian phase)*	Photic phase shifting via ipRGC–SCN signaling	PD: reduced excessive daytime sleepiness, improved sleep quality; dementia: inconsistent effects on sleep, behavior and cognition	PD: small RCT (low to moderate) [82]; dementia: heterogeneous findings across trials (low)
*Melatonin (immediate-release, timed)*	Chronobiotic signal/phase-shifting agent; sleep-promoting effect	RBD: fewer dream-enactment behaviors; insomnia: modest effect on sleep onset; AD: no consistent cognitive benefit	Conditional guideline recommendation for RBD; small RCTs; low certainty in PD/AD [46,85]
*Time-restricted eating*	Feeding-related entrainment of peripheral clocks; nutrient-sensing pathways	Metabolic improvement in metabolic syndrome or prediabetes; preliminary cognitive signal in MCI	Indirect (metabolic RCTs); one pilot RCT in MCI; no trials in AD/PD; preclinical in AD mice [61,62,120,121]
*Moderate-to-vigorous physical activity*	Non-photic zeitgeber	Lower mortality; higher rest–activity amplitude	Observational cohorts; no circadian-outcome RCTs in AD/PD [13,93]
*Evening light reduction/blue-light filters*	Limits melatonin suppression and phase delay	Inconsistent effects on sleep quality	Very low certainty for filtering lenses; no AD/PD trials [92]
*Clock-targeting drugs (REV-ERB agonists, CRY stabilizers)*	Direct modulation of the TTFL	Altered gene expression and reduced amyloid burden in experimental models	Preclinical only [117,118]

**Table 4:** Chrononutritional strategies: mechanisms, relevance and type of supporting evidence.

*Strategy*	Biochemical Mechanism	Relevance to Circadian, Sleep and Neurodegenerative Outcomes	Type of Evidence
*Meal timing aligned to the active phase*	Feeding entrains hepatic, adipose, muscle and gut clocks; avoids nocturnal metabolic load	May improve internal synchrony and glucose tolerance; neurological outcomes not tested	Controlled human experiments in healthy adults; animal studies [16,22,77]
*Time-restricted eating/intermittent fasting*	Lower insulin and mTORC1, higher AMPK, ketogenesis and autophagy	Metabolic benefit; memory and amyloid improvement in AD mice; short-term cognitive signal in MCI; risk of undernutrition in frail patients	Preclinical and indirect human RCTs; one pilot RCT in MCI; none in AD/PD [61,62,123,129]
*NAD^+^ precursors (niacin, nicotinamide, NR, NMN)*	Support of the CLOCK/BMAL1–NAMPT–NAD^+^–SIRT1 loop and mitochondrial metabolism	Target engagement in PD; no clinical benefit in a phase 3 trial in early PD; no cognitive benefit in MCI	Direct RCTs: phase 1 positive for biochemical endpoints, phase 3 negative [14,135,136]
*Protein redistribution in levodopa-treated PD*	Less competition of dietary amino acids with levodopa transport	Improved daytime motor response in selected patients with fluctuations; total protein intake must be preserved	Small trials and clinical guideline [43]
*Tryptophan- and melatonin-containing foods*	Substrate for serotonin/melatonin synthesis; multiple bioactive compounds	Short-term improvements in selected sleep outcomes	Small, short trials in insomnia; no AD/PD data [137,138]
*Magnesium, omega-3 fatty acids, polyphenols*	NMDA/GABA modulation; pro-resolving mediators; sirtuin/AMPK engagement	Magnesium: subjective sleep improvement in older adults with insomnia; DHA and resveratrol: no clinical efficacy in AD	Small RCT (magnesium); large negative RCT (DHA); phase 2 trial without efficacy (resveratrol) [139,140,141]
*Fermentable fiber/microbiota-directed diet*	Short-chain fatty acids modulate metabolism, immunity and the gut–brain axis	Hypothesized support of circadian and neuro-immune homeostasis	Preclinical and observational only [7,134]

**Table 5:** Representative human nutritional intervention studies: exposure, outcomes and clinical directness.

Study/Population/Design	Exposure and Duration	Principal Findings	Evidence Appraisal/Relevance
Sutton et al.; eight men with prediabetes; randomized crossover [120].	Six-hour early eating window; dinner before 15:00; five weeks per condition; weight maintained.	Selected insulin sensitivity, blood pressure and oxidative-stress measures improved.	Controlled metabolic proof of concept; very small male sample; no neurological endpoints.
TIMET; metabolic syndrome; randomized trial, 108 completers [121].	Personalized 8–10-h TRE plus standard care versus standard care; three months.	HbA1c between-group difference −0.10 percentage points (95% CI −0.19 to −0.003).	Modest metabolic effect; indirect to AD/PD; does not isolate a brain-clock mechanism.
Wang et al.; MCI; pilot randomized trial, n = 46 [62].	15:9 TRE versus usual intake; 12 weeks.	Improvement in selected short-term cognitive scores.	Preliminary direct MCI evidence; not confirmed AD; no progression or dementia-prevention endpoint.
NADPARK; early untreated PD; phase 1 randomized trial, n = 30 [135].	NR 1000 mg/day versus placebo; 30 days.	Variable cerebral NAD+ increase and metabolic/inflammatory changes.	Direct PD target engagement; exploratory clinical findings; too short to establish disease modification.
NOPARK; early PD; phase 3 randomized trial, n = 410; primary analysis n = 393 [14].	NR 500 mg twice daily versus placebo; 52 weeks.	MDS-UPDRS I–III adjusted difference 2.72 points (95% CI 0.47–4.98), favoring placebo; NMSS also favored placebo.	Strongest direct PD efficacy evidence here: no support for disease-modifying NR. Score differences below reported clinically important thresholds.
Orr et al.; MCI; pilot randomized trial, n = 20 [136].	NR escalated to 1000 mg/day; ten weeks.	Blood NAD+ increased; no cognitive benefit. Exploratory blood-flow results require multiplicity caution.	Biochemical response without established neurological efficacy; small trial.
NR cognitive/biomarker trial; SCD/MCI; randomized crossover, 46 randomized, 37 completed [143].	NR 1000 mg/day; eight-week active treatment.	No significant primary cognitive benefit; exploratory plasma pTau217 difference.	Biomarker signal requires replication; not evidence of clinical disease modification.
Yoshino et al.; 25 postmenopausal women with prediabetes; randomized trial [144].	NMN 250 mg/day versus placebo; ten weeks.	Improved skeletal-muscle insulin sensitivity.	Indirect peripheral metabolic evidence; no AD/PD or cognitive efficacy claim.
Abbasi et al.; primary insomnia in older adults; randomized trial, n = 46 [139].	Magnesium 500 mg/day versus placebo; eight weeks.	Selected subjective sleep measures improved.	Small, short study outside AD/PD; does not establish circadian realignment.
Hudson et al.; chronic insomnia; three-arm study, 57 recruited, 49 completed [137].	Food-source or pharmaceutical tryptophan, approximately 250 mg with carbohydrate, versus carbohydrate; three weeks.	Selected sleep improvements with tryptophan-containing preparations.	Preparation-specific, short-term evidence; no neurodegenerative population.
Losso et al.; insomnia; small randomized crossover, eight completers [138].	Tart cherry juice 240 mL twice daily; two weeks per condition.	Some sleep outcomes improved; sleep-apnea exclusions reduced sample.	Very low precision; multiple bioactives; effect cannot be attributed specifically to melatonin.
Quinn et al.; mild-to-moderate AD; randomized trial, n = 402 [140].	DHA 2 g/day versus placebo; 18 months.	No slowing of overall cognitive or functional decline.	Direct AD trial; negative overall efficacy result; no individualized circadian dosing.
Turner et al.; AD; phase 2 randomized trial, n = 119 [141].	Resveratrol 500 mg/day escalated to 1000 mg twice daily; 52 weeks.	Some biomarker trajectories changed; gastrointestinal effects and weight loss relevant.	No established disease-modifying efficacy; biomarker changes are not proof of benefit.

Abbreviations: AD, Alzheimer’s disease; PD, Parkinson’s disease; MCI, mild cognitive impairment; SCD, subjective cognitive decline; TRE, time-restricted eating; NR, nicotinamide riboside; NMN, nicotinamide mononucleotide.

## Data Availability

No new data were created or analyzed in this study. Data sharing is not applicable to this article.
